# Preference-based scoring algorithm to estimate societal utilities based on the patient-reported experience of cognitive impairment in schizophrenia (PRECIS) instrument

**DOI:** 10.1007/s11136-026-04308-7

**Published:** 2026-06-15

**Authors:** Martine Hoogendoorn, Irene Santi, Anna-Katrine Sussex, Daniel Aggio, Jenny Wang, Andrew Lloyd

**Affiliations:** 1 Institute for Medical Technology Assessment, Erasmus University Rotterdam, Burgemeester Oudlaan 50, 3062 PA Rotterdam, The Netherlands; 2 Erasmus School of Health Policy and Management, Erasmus University Rotterdam, Rotterdam, The Netherlands; 3 Acaster Lloyd, London, UK; 4 Boehringer Ingelheim International GmbH, Ingelheim, Germany

**Keywords:** Schizophrenia, Utility, Cognitive impairment, PRECIS, Cost-effectiveness

## Abstract

**Purpose:**

Generic measures of health-related quality of life (HRQoL), such as the EQ-5D, may inadequately capture the impact of cognitive impairment in schizophrenia, resulting in incorrect QALY estimates in economic evaluations. This study aimed to obtain health utilities by valuing key items of the Patient-Reported Experience of Cognitive Impairment in Schizophrenia (PRECIS) instrument.

**Methods:**

First, expert interviews and psychometric analysis of the 28-item version of the PRECIS were performed to identify the best-performing and most relevant items. Second, health states based on the four selected PRECIS items representing memory, communication, executive function and attention (each with five levels of severity) were valued by the UK general public through a discrete choice experiment (DCE) survey and composite time trade-off (cTTO) interviews. To estimate a utility scoring algorithm, the DCE data were analyzed using mixed logit models and rescaled onto a 0–1 (dead to full health) utility scale using the cTTO results.

**Results:**

The cTTO utility for the best health state (level 1, “not at all hard”, across all domains) had a mean utility of 0.992 (SD 0.038) while the worst state (level 5, “very hard”, across domains) had a mean utility of 0.292 (SD 0.479). All domains significantly predicted larger utility decrements with increasing impairment severity.

**Conclusion:**

This study demonstrated the feasibility of quantifying health utility values for patient-reported cognitive impairment domains using the validated PRECIS instrument. The resulting utilities can capture the HRQoL impact of cognitive impairment, supporting more accurate future economic evaluations of therapies targeting cognitive function in schizophrenia.

**Supplementary Information:**

The online version contains supplementary material available at https://doi.org/10.1007/s11136-026-04308-7.

## Abstract (plain language summary)

New treatments are under development that aim to improve cognitive impairment in schizophrenia. Studies estimating the costs and benefits of these treatments, used for reimbursement decisions, often underestimate the benefits because generic questionnaires used to measure the quality of life of patients, don’t include questions on cognition. This study addresses how to more accurately capture and quantify the impact of cognitive impairment in schizophrenia on quality of life to make accurate reimbursement decisions. Based on an existing questionnaire that assesses the patient-reported cognitive impairment in schizophrenia (PRECIS) the most important aspects of cognitive impairment for patients were selected. The UK general public was asked to value the importance of the selected four items and their answer options, Results showed that the selected aspects, memory, communication, executive function, and attention all had an impact on quality of life with worse quality of life outcomes when the level of impairment was larger. This study contributed to a method for more accurate estimates of the benefits of new treatments aiming to improve cognitive impairment in schizophrenia.

**Supplementary Information:**

The online version contains supplementary material available at https://doi.org/10.1007/s11136-026-04308-7.

## Introduction

Schizophrenia is a heterogenous mental health disorder, which is characterized by hallucinations, delusions, alongside disordered thoughts and behavior [1, 2]. Cognitive impairment is common among patients with schizophrenia, and this can significantly impact their daily functioning, independence, and employability [3, 4]. Current medications for schizophrenia primarily address positive symptoms such as delusions and hallucinations but have limited efficacy in improving cognitive deficits. However emerging treatments are being evaluated to determine if they can improve cognitive function. As such treatments undergo regulatory review, there is a need in some countries to evaluate their cost-effectiveness.

Cost-effectiveness analyses typically consider health benefits in terms of quality-adjusted life-years (QALYs), which combines life expectancy with health-related quality of life (HRQoL). To accurately assess HRQoL it is important to measure the dimensions of health that are affected by the disease. The commonly used EQ-5D instrument is a generic measure of HRQoL which assesses mobility, self-care, usual activities, pain/discomfort, and anxiety/depression. There is no dimension that specifically measures cognitive functioning [5]. If the EQ-5D cannot measure cognitive impairment accurately then it may underestimate the value of treatments that target cognition, such as the new treatments in schizophrenia. The limitation of the EQ-5D with respect to measuring cognitive aspects has been recognized as early as 1999 [5]. The evidence for performance of the EQ-5D in schizophrenia has been mixed, with studies showing good known-group validity but only weak-to-moderate correlations of the EQ-5D dimensions with symptom severity [6, 7].

One potential solution to address this limitation and to obtain health utilities that do include the impact of cognition, is to directly value measures that do assess cognitive impairment. Cognitive impairment is a multidimensional construct which includes for example attention, memory, decision making and language. By breaking cognition into sub-domains, unique utility values for each domain can be estimated. Utilizing existing validated instruments in schizophrenia to measure multidimensional cognitive impairment also allows us to leverage previous research data to support decision making.

Several instruments are available to measure cognitive impairment in schizophrenia. The Patient Reported Experience of Cognitive Impairment in Schizophrenia (PRECIS) captures patient-reported experiences of cognitive impairment [8, 9], complementing more objective measures, such as the MATRICs Consensus Cognitive Battery (MCCB) and the Schizophrenia Cognition Rating Scale (SCoRS) [10–13]. Health utilities derived from the interviewer-based SCoRS have been previously estimated [14]. The 28-item version of the PRECIS was found to be moderately correlated with functioning instruments, such as the SCoRS, but only weakly correlated with the MCCB, reflecting that the PRECIS captures unique, patient-experienced aspects of cognitive impairment that are not fully represented by performance-based cognitive measures [9].

The present study aimed to estimate valuation weights for items of the PRECIS instrument to be able to assign utility weights to aspects of cognitive impairment based on patients’ experience. The resulting health utilities can be used to improve health benefit estimates in future economic evaluations of treatment options targeting cognitive impairment in schizophrenia.

## Methods

The study consisted of two parts. First, extensive psychometric analyses were performed in combination with expert interviews to select the best performing and most relevant items of the PRECIS. Second, health states based on the selected items were included in a valuation study and results were analyzed to obtain a scoring algorithm for estimating health utilities. The 28-item version of the PRECIS instrument includes a total score and six cognitive domain sub-scores: memory, communication, self-control, executive function, attention, and sharp thinking. Two additional items assessing degree of bother are not included in any domain or total score [8]. Each item is rated on a 5-point Likert scale, with higher scores corresponding to more severe patient impairment (1 = not at all/not at all hard, 2 = a little bit/a little bit hard, 3 = somewhat/somewhat hard, 4 = quite a bit/quite hard, 5 = very much/very hard).

### Psychometric analyses of the PRECIS

Baseline PRECIS data of patients participating in a multi-center phase 2 randomized clinical trial (NCT02832037) were used to perform psychometric analyses on the PRECIS instrument [15]. The total trial population consisted of *n* = 509 patients, but the PRECIS instrument had been administered to participants from study sites in the US only (*n* = 215). The characteristics of the patients that completed the PRECIS were comparable to the total trial population except for ethnicity. Patients were on average 38 years old, 71% were male and 54% had black or African American ethnicity. Descriptive statistics were performed for each item of the PRECIS, including the frequency distribution of item responses, floor and ceiling effects, and percentage of missing responses. Internal consistency, i.e. whether items within a subscale measure the same concept, was assessed for the items within each of the PRECIS’s six domains of cognition with Cronbach’s alpha (α) and item-total domain score correlation (with the item deleted) (Online Supplementary Material, Appendix I). Correlation coefficients (Spearman's Rho) between PRECIS items and the EQ-5D visual analogue scale provided insight into which items might be associated with HRQoL (Online Supplementary material, Appendix I). The current study also considered results from previously conducted exploratory factor analysis (EFA) and item response theory (IRT) analysis using the same baseline PRECIS data [9, 16]. The performance of the items in each of the six PRECIS factors/domains was evaluated by fitting the Samejima’s graded response model (GRM). For the current analysis the item slope obtained from the model was used to assess the strength of the relationship to the construct of interest. More details are reported in the study conducted by Lenderking et al. [9].

### Expert advice

Expert input from one health economics specialist and three clinical specialists also guided the item selection process. The four experts reviewed information from the psychometric analysis, considered clinical relevance of items and provided feedback on which aspects of cognitive impairment have the most impact on quality of life. Furthermore, the potential use of the items within vignettes was carefully considered to ensure the items were appropriate and understandable for a general population audience without significant rephrasing or explanation. A maximum up to 6 items was considered feasible for the valuation study to aid respondent comprehension and avoid cognitive burden.

### Final PRECIS item selection

The final item selection was based on the psychometric analyses for ceiling effects, correlation with the VAS, factor loading of the item in the EPA, and the IRT analysis item slope and expert opinion (See Online Supplementary material, Appendix II for the final decision matrix). For each type of psychometric analysis, the results for each item were normalized using min–max scaling, ensuring that the scores for the 26 items ranged from 0 to 1. These normalized scores were then summed, and the item with the highest normalized score within each domain was identified as the best-performing item based on the psychometric analysis. Normalized sum scores for the psychometric analyses were highest for item 4 (memory), 10 (communication), 12 (self-control), 16 (executive function), 18 (attention) and 24 (sharpness of thinking). Expert interviews showed that the four experts ranked the domains Self-control and Sharp thinking as less important than the other domains. Selection of the most important items within the domains by the experts was in accordance with the psychometric analysis for the domains Memory and Communication. Because the six domains of the PRECIS are inherently interrelated and do not function as discrete domains, including one item per domain was not considered necessary. Based on the psychometric analyses and expert opinion to evaluate the suitability of the items collectively, the following four items representing the domains memory, communication, executive function and attention were selected for the valuation study:

- “Overall, in the past week remembering what someone else was saying was not at all hard/a little bit hard/somewhat hard/quite hard/very hard”.
- “Overall, in the past week finding words to say what I mean was not at all hard/a little bit hard/somewhat hard/quite hard/very hard”.
- “Overall, in the past week coming up with solutions to problems was not at all hard/a little bit hard/somewhat hard/quite hard/very hard”.
- “Overall, in the past week my mind drifted off when I wanted to pay attention not at all/a little bit/somewhat/quite a bit/very much”.

### Valuation study

Following item selection, a discrete choice experiment (DCE) survey was designed using health state descriptions based on the four selected PRECIS items and their response options. Health state 2324, for example, represents a patient that scores “a little bit hard” (level 2) on the Memory item, “somewhat hard” (level 3) on Communication, “a little bit hard” (level 2) on Executive Function, and “quite a bit” (level 4) on Attention. The initial DCE survey underwent cognitive debriefing interviews with five UK general population participants to ensure clarity and appropriateness. Based on this feedback, the DCE was finalized. Subsequently, a larger UK population sample completed the online DCE, indicating their preferences between pairs of health states. A subset of responders then participated in composite time-trade off (cTTO) interviews to assign utility values for selected PRECIS-based health states. Finally, regression modelling was conducted using DCE data rescaled with cTTO data to estimate a scoring algorithm for health utilities based on the PRECIS items.

### Sample size

A power calculation was performed using the following assumptions: use of orthogonal design (a priori assumption of six items, five levels), linear disutility per item level (0.03), standard deviation (SD) of disutility per health state (0.2), random draw for each simulated health state value is a truncated normal distribution with upper level is 1 and lower level is zero, random effects panel regression to estimate a 25 parameter model (four dummies with six parameters + constant) and 100 datasets generated for each sample size (100 to 1000) [17]. The power analysis showed that a sample size of *N* = 600 was considered sufficient for a valuation study with four items. Changes in assumptions on the size of the SD of disutility and non-linearity of the disutility per item did not result in different sample sizes.

### Data collection

Members of the UK general population were recruited via a specialist online research recruitment platform (Prolific, https://www.prolific.com/) for three phases of the study: qualitative pilot interviews, the online DCE survey, and the cTTO interviews. Quotas were set to ensure an approximate match to the demographic profile of the UK general population based on 2021 census data (age, sex, ethnicity) [18]. Participants were eligible if they were aged 18–70 years old, living in the UK, able to read and speak in English, able to give informed consent to participate in an interview/survey, had access to a webcam and microphone (built-in or external) for the interview, and access to a laptop or desktop computer (not mobile phone or tablet). Eligible participants were given detailed study information and asked to provide informed consent to participate through yes/no responses to statements integrated into an online survey link. Participants received paid compensation for participating in the study, £40 (GBP) for the qualitative pilot interview, £6 for the online DCE survey and/or £40 for the online cTTO interview.

### Design and conduct of the DCE survey

DCE is a stated-preference method that can be used to elicit individual’s preferences between different aspects of health states [19]. The aim of this DCE was to quantify preferences regarding cognitive impairment domains represented by the four selected PRECIS items representing memory, communication, executive function and attention. Based on the selected items (attributes) and answer options (levels), a D-efficient experimental design was created with 40 unique choice tasks divided into four blocks, each containing ten tasks. Each participant completed one randomly assigned block of 10 experimental tasks. The design was generated using the software NGene (Version 1.2) [20], incorporating directional priors capturing information about the expected direction of preferences (e.g., positive preference for less severe impairment) to minimize the risk of dominant alternatives.

**Fig. 1:**
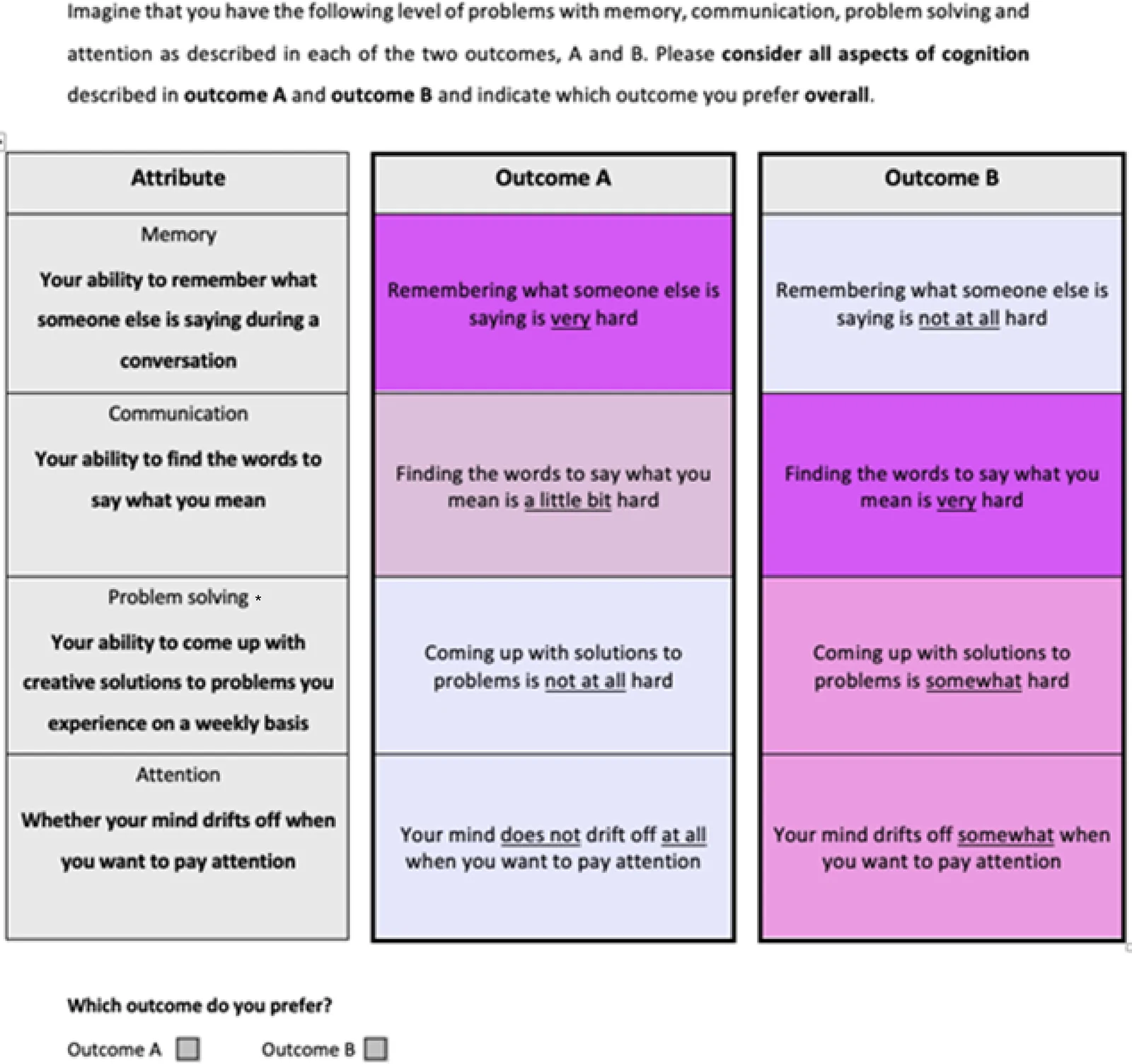
Example of a choice task in the discrete choice experiment (DCE). * To address cognitive debriefing interview feedback, the domain “executive function” was presented in the survey as “problem solving”, which was also better in line with the original PRECIS item

After a brief introduction of the attributes and levels and a set of comprehension questions to check understanding, participants were presented with a series of choice tasks (Fig. 1 as an example). Each paired description in a choice task was varied based on the experimental design generated. Each participant also completed two additional choice tasks known as dominance and repeated-choice tests as indicators of participant attention and understanding. Participants then completed demographic questions and the EQ-5D-5L.

### Design and conduct of the cTTO interviews

Time-trade-off (TTO) is a standardized interview method for valuing health states, which is used to generate utility weights for selected descriptions of health [21, 22]. Structured TTO interviews which allow for health states to be valued as worse than dead, known as composite TTO (cTTO) [23], were conducted to elicit utility valuations for the best and worst health states, as well as three other moderately severe health states. The best health state was described by the best possible response option for each cognition item (Not at all hard). Similarly, the worst health state was described by the worst possible response option for each item (Very hard). The three moderate health states were described using the same items with a mixture of response option severity to capture the moderate response levels (e.g., a little bit hard, quite hard). Interviews were conducted online via a video-calling platform by experienced interviewers using a structured interview guide.

During the cTTO interviews, participants were asked to rank the five health states in order of preference (from most preferable to least preferable) on a visual analogue scale (VAS) from 0–100, where 100 represents full health and 0 represents worst possible health. After completing the ranking, participants were asked to imagine that they were the person affected by each of the presented health states. For each health state, they were asked to choose whether they preferred to live in the health state for 10 years followed by death or to live in [10 – X] years of full health. They could also indicate that the two options were equal. The process incorporates a ‘ping-pong’ approach with the number of years of life in ‘full health’ traded back and forth between higher (e.g. 9 years) and lower (e.g. 1 year) values that iteratively narrow in 6-month increments to the point of indifference. The point of indifference is where the participant cannot differentiate between the reduced number of years in ‘full health’ versus 10 years in a study or practice health state. If a health state was considered to be worse than dead (i.e. 0 years in full health is preferred over 10 years of life in a health state), an adaptation of the TTO task, known as lead-time TTO was used to determine the extent to which the participant values a health state as worse than death. In lead-time TTO, participants were asked whether they would prefer to live for 10 years in full health followed by 10 years in the health state, or to live for X years of full health (where X < 10). The interview then followed the same ‘ping-pong’ approach until a point of indifference was reached. This lead-time procedure allows the participant to trade more years of life to determine how much worse than dead they consider the health state to be. Participants completed the cTTO task for two practice health states, based on EQ-5D dimensions, followed by the five health states. The health state vignettes were presented in four different order sets to reduce ordering effect.

### Estimating the PRECIS scoring algorithm

The DCE data were analyzed based on the random utility framework using multinomial logit (MNL) and mixed logit (MXL) models. The final model selection was based on statistical performance across the two models using goodness of fit statistics such as the Akaike information criterion (AIC), Bayesian information criterion (BIC), and McFadden adjusted R^2^. The likelihood ratio (LR) test was used to determine whether the improvement in fit from a more complex model is statistically significant, guiding the selection of a model that justifies its added complexity [24–27]. To facilitate the use of the ordinal DCE preference results in economic evaluations, results needed to be rescaled between 0 (representing death) and 1 (representing full health), using cTTO-data anchors [28].

The estimated coefficients from the DCE model were normalized onto the full health-dead scale by dividing the coefficient on each level by a rescaling parameter θ, calculated as follow:

1 $$ \theta = \frac{{U_{DCE} (WS) - U_{TTO} (BS)}}{{U_{TTO} (WS) - U_{TTO} (BS)}} $$

where $U_{TTO} \left( {WS} \right)$ and $U_{TTO} \left( {BS} \right)$ are the observed mean utility estimates for the worst and best health states in the TTO valuation tasks, and $U_{DCE} \left( {WS} \right)$ is $U_{TTO} \left( {BS} \right)$ plus the sum of the DCE coefficients for the worst level of each attribute, i.e. the most severe impairment in each PRECIS-defined domain of cognition. Since by design, $U_{DCE} \left( {BS} \right)$ is set to zero and the best state on a disease-specific measure does not necessarily represent full health, an adjustment was included in the formula to align the scales, setting $U_{DCE} \left( {BS} \right)$ equal to $U_{TTO} \left( {BS} \right)$.

The mean utility value for a health state $V_{j}$ is calculated using the rescaled coefficient estimates from the DCE as follow:

2 $$ V_{j} = U_{TTO} \left( {BS} \right) + \mathop \sum \limits_{j = 1}^{4} x_{j} $$

where $x_{j}$ are the rescaled coefficients representing the disutility of each attribute level in the health state.

## Results

### Valuation study

The DCE survey was completed by *n* = 500 participants, recruited to reflect the UK general population in terms of regional distribution, age, sex, and ethnicity, with a slightly higher representation of individuals with higher education levels (Table 1). The EQ-5D-5L scores, derived using the 5L-3L crosswalk of van Hout et al. [32], showed an average health-related quality of life score of 0.801, somewhat lower than the UK general population norms, while the VAS mean score of 75.11 suggests slightly lower self-perceived health. Overall, the health status in this sample aligned closely with national averages, making it representative of the general population. Anxiety was the most frequent self-reported health condition, affecting 36.4% of participants, followed by depression at 27.6%.

**Table 1:** Study sample and UK general population sociodemographic characteristics (*N* = 500)

Characteristic	Total *N* = 500	UK population [29]
UK area (*n*,%)		
Wales	17 (3.4%)	4.5%
England	425 (85.0%)	84%
Northern Ireland	12 (2.4%)	2.8%
Scotland	46 (9.2%)	8.2%
Age (years), mean (SD)	41.7 (12.0)	40.9
Males (*n*,%)	250 (50.0%)	49.2%
Ethnicity (*n*,%)		
Asian	48 (9.6%)	9.3%
Black	19 (3.8%)	4.0%
Mixed/other	25 (5.0%)	5.0%
White	408 (81.6%)	81.7%
Education (*n*,%)		
No formal qualifications	10 (2.0%)	
Left school at age 16 with qualifications	40 (8.0%)	
Left school at age 18 with qualifications	88 (17.6%)	
Technical/ vocational qualifications	26 (5.2%)	33.8% [30]
Completed university (Bachelor’s degree or above)	327 (65.4%)	
Other	8 (1.6%)	
Prefer not to answer	1 (0.2%)	
EQ-5D-5L utility score, mean (SD)	0.801 (0.196)	0.856 [31]
EQ-5D VAS score, mean (SD)	75.11 (17.25)	82.8

*SD* Standard deviation, *Q*1 1st quartile (25th percentile), *Q*3 3rd quartile (75th percentile)

The DCE survey included tasks on comprehension, attention and dominance as data quality checks. In total, 36 out of 500 participants failed one of these three tasks. The respondents who failed the tests were slightly younger compared to the respondents who did not fail the tests (38.8 versus 41.9 years), were more likely to be employed full-time (75% versus 68.3%) and less likely to have a university degree (52.8% versus 65.4%).

Results for the MNL and MXL models (Online Supplementary Material, Appendix III), estimated based on the DCE data, showed that all attributes (memory, communication, executive function, and attention) were significant predictors of participants’ preferences between health state descriptions. The MXL model showed stronger performance (higher adjusted Pseudo R^2^ and better fit with lower AIC value) than the MNL model and was considered the preferred model. A sensitivity analysis excluding responders who failed at least one of the comprehension, attention or dominance tasks showed that findings were consistent with the primary analysis.

Table 2 shows the results of the cTTO completed by a sub-sample of *n* = 100 respondents, also demographically representative of the UK general public. The cTTO utility value and the VAS score generally agree on the relative ordering of health states, defined by the PRECIS items and their response options, but differ in the absolute value/score of the three intermediate states (Table 2). For health state 2324 for example, the cTTO value was 0.827 and the VAS score was 59.1. In contrast, health state *4432*, reflecting more pronounced difficulties across domains—shows lower mean cTTO (0.728) and VAS (49.5) values. This pattern illustrates that both the TTO as well as the VAS capture the expected decline in perceived health as cognitive impairment increases.

**Table 2:** Distribution of cTTO values and VAS scores by health state.

	cTTO (0–1 scale)			VAS (0–100 scale)		
Health State (level)*	Mean (SD)	Median (Q1, Q3)	Range (Min–Max)	Mean (SD)	Median (Q1, Q3)	Range (Min–Max)
1111 (Best)	0.992 (0.038)	1.000 (1.000, 1.000)	0.700–1.000	96.3 (5.6)	100.0 (95.0, 100.0)	75.0–100.0
2324	0.827 (0.16)	0.875 (0.750, 0.950)	0.200–1.000	59.1 (15.7)	60.0 (50.0, 70.0)	25.0–90.0
3244	0.762 (0.22)	0.800 (0.700, 0.931)	0.075–1.000	51.7 (15.6)	50.0 (40.0, 60.0)	20.0–90.0
4432	0.728 (0.23)	0.800 (0.600, 0.900)	0.075–0.975	49.5 (15.4)	49.0 (40.0, 60.0)	15.0–90.0
5555 (Worst)	0.292 (0.479)	0.200 (0.050, 0.681)	− 1.000–0.975	27.1 (15.8)	22.5 (16.5, 35.0)	0.0–90.0

SD, Standard deviation; Q1, 1st quartile (25th percentile); Q3, 3rd quartile (75th percentile) *Levels of 1 “not at all hard”, 2 “a little bit hard”, 3 “somewhat hard”, 4 “quite hard”/ “quite a bit” to 5 “very hard”/ “very much” in order of Memory, Communication, Executive Function, and Attention, respectively

To estimate a utility scoring algorithm, the regression coefficients from the MXL model were rescaled using the rescaling parameter θ, which was calculated to be 15.28. The rescaled coefficients from the MXL model are reported in Table 3. All attributes (memory, communication, executive function, and attention) were significant predictors of general public perceptions of health states, with increasing levels of impairment resulting in larger utility decrements. Decrements for the attention attribute were in general smaller than for the other three attributes.

**Table 3:** DCE Model Estimates—rescaled results

	Rescaled mixed model results (rescaled onto 0–1 utility scale using cTTO data)		
	Coefficient	Standard error	*P*-value
ASC* – Choice 2	− 0.0024	0.0037	0.515
Memory (2)	− 0.0376	0.0075	< 0.001
Memory (3)	− 0.0567	0.0069	< 0.001
Memory (4)	− 0.1121	0.0084	< 0.001
Memory (5)	− 0.1866	0.0116	< 0.001
Communication (2)	− 0.0074	0.0078	0.314
Communication (3)	− 0.0521	0.0080	< 0.001
Communication (4)	− 0.0790	0.0084	< 0.001
Communication (5)	− 0.1940	0.0122	< 0.001
Executive Function (2)	− 0.0541	0.0073	0.006
Executive Function (3)	− 0.0819	0.0077	0.005
Executive Function (4)	− 0.1211	0.0082	< 0.001
Executive Function (5)	− 0.1891	0.0122	< 0.001
Attention (2)	− 0.0212	0.0078	0.006
Attention (3)	− 0.0224	0.0080	0.005
Attention (4)	− 0.0590	0.0084	< 0.001
Attention (5)	− 0.1303	0.0122	< 0.001

Level 1 of each attribute is the reference level “not at all/not at all hard”

(2) a little bit/a little bit hard, (3) somewhat/somewhat hard, (4) quite a bit/quite hard, (5) very much/very hard

ASC, Alternative-Specific Constant *The model includes the ASC. This controls for any bias in selecting alternatives based on their position on the left or right side of the screen. This constant is particularly important in the present study because the order of the alternatives was not randomized within each choice task. The ASC captures the effect of presentation rather than the characteristics of the health state. Therefore, it is excluded when computing the utility value

These rescaled coefficients can be used to estimate the utility of cognitive impairment health states defined using the selected four PRECIS items. An example of this is shown in Table 4 for a patient with health state 3234 (memory level 3, communication level 2, executive function level 3 and attention level 4). The final utility estimate of the example 0.787 is obtained by subtracting from the value of the best health state (0.992) the sum of the decrements associated with the four domains (− 0.0567 − 0.0074 − 0.0819 − 0.0590).

**Table 4:** Example of utility calculation based on the estimated utility scoring algorithm for the PRECIS

Domain	Patient response on the PRECIS items	Utility estimation compared to best health state* (0.992)
Memory	Overall, in the past week remembering what someone else was saying was somewhat hard (level 3)	− 0.0567
Communication	Overall, in the past week finding words to say what I mean was a little bit hard (level 2)	− 0.0074
Executive function	Overall, in the past week coming up with solutions to problems somewhat hard (level 3)	− 0.0819
Attention	Overall, in the past week my mind drifted off when I wanted to pay attention, quite a bit (level 4)	− 0.0590
Final utility estimate		0.787

^*^Best health state: levels of 1 “not at all hard” for all domains of Memory, Communication, Executive Function, and Attention

## Discussion

This study demonstrates the feasibility of using the PRECIS instrument to estimate utilities associated with cognitive impairment in schizophrenia. The utility values for the worst and best cognitive health states defined by the selected PRECIS items were estimated to be 0.292 and 0.992, respectively. Based on the rescaled parameters of the DCE mixed model, the utility value for all different response level combinations of the four items from the PRECIS can be estimated. This final algorithm facilitates a more accurate representation of the impact of cognitive health on quality of life than currently reflected in the EQ-5D and can be used in cost-utility analyses. The National Institute for Health and Care Excellence (NICE)’s manual [33] recognizes that some important aspects of health lie outside of the descriptive system of the EQ-5D, such as vision and hearing and cognition and emphasizes the necessity of measuring and valuing health effects in a way that fully reflects the impact of interventions on relevant dimensions of health. The current study addresses that limitation by developing preference-based utilities specific to cognitive impairments [6], which makes it possible to present both the QALYs based on the EQ-5D as well as the QALYs based on the PRECIS instrument to show the impact of the intervention when including relevant cognitive aspects.

There are a number of significant strengths associated with this study design. First, this valuation study was performed using an existing instrument to measure cognitive impairment in schizophrenia. The 28-item PRECIS instrument is a valuable patient-reported outcome in itself. Next to that, the algorithm estimated in the current study based on the four best performing items can be applied to estimate utilities for all studies that include the PRECIS.. The selection of items for valuation was informed by extensive psychometric analysis and expert opinion, ensuring that the most relevant aspects of cognitive functioning were captured while balancing feasibility. A second, significant strength is the application of a rigorous, mixed-methods approach to estimate utilities. The use of a DCE combined with cTTO ensured that the resulting utility estimates are both robust and interpretable on the full health-to-dead scale. This methodological rigor enhances the applicability of the findings in QALY-based evaluations. A third strength is the use of a general population sample for utility estimation, which aligns with best practice in economic evaluation. The study found that the general public could assess and value differences in cognitive function. The resulting scoring algorithm provides a tool for directly applying these utilities to clinical trial data, facilitating the assessment of interventions targeting cognitive impairments.

The study has limitations that warrant consideration. First, the self-reported nature of PRECIS, is useful for capturing the patient perspective. But with more severe loss of function the patient will not be able to reliably report their cognitive status. This may limit the use of the measure and would mean that assessment could switch to a proxy report. Second, utilities derived exclusively from cognitive impairment do not represent the full spectrum of HRQoL impacts associated with schizophrenia. These data could be considered as a way of weighting existing utilities in schizophrenia where patients also experience cognitive impairment. Different methods exist for doing this [34]. Therefore, the PRECIS-derived utilities could be applied alongside broader HRQoL measures to comprehensively assess treatment impacts. Third, utilities were derived based on UK societal preferences. Although generic measures like EQ-5D typically exhibit limited variation across culturally similar countries, the extent to which cultural differences affect perception and valuation of cognitive impairments (especially in the context of schizophrenia) remains uncertain. Thus, future cross-cultural validation studies are recommended to ensure broader applicability of the derived utilities. Fourth, for feasibility, the valuation focused on a limited set of PRECIS items. Consequently, certain clinically or economically significant aspects of cognitive impairment might have been omitted. This selective approach, while methodologically necessary, warrants careful consideration in future research and clinical applications. Although an expert panel of clinical and quality of life experts was consulted to select the most important aspects of cognitive impairment, future studies should also include patient organizations and other stakeholders. Lastly, the observed ceiling effects in some PRECIS items due to the relatively low levels of impairment observed in the Phase-2 clinical trial may have impacted the results of the psychometric analysis and therefore the item selection. Responsiveness of the new algorithm to changes using available trial data was therefore also difficult to assess, because the room for improvement in patients in the trial was limited. Future research should focus on validating PRECIS utilities in diverse populations of people with schizophrenia, especially more severe or different cultural populations.

When comparing the approach in the current study with the approach in the EQ-VT 2.0 protocol [35] a couple of differences can be noted. In the EQ-VT protocol each respondent completes a block of cTTO tasks followed by a block of DCE choice tasks, while in the current study, a sub-sample of those completing the DCE tasks completed the cTTO interviews. In the current study blocks of health states for the DCE tasks were used like in the EQ-VT protocol. Using blocks for the cTTO phase in the current study was deemed unnecessary due to the small number of health states being valued by cTTO.”

Besides direct valuation of cognitive measures such as the PRECIS as performed in the current study, other solutions are proposed to address the limitation of EQ-5D with respect to measuring cognition. Several studies explored the option of developing a “bolt-on”, an additional item, to the EQ-5D for cognition [5, 36, 37]. The main challenge of bolt-ons is that any valuation study of a bolt-on involves a re-evaluation of the core set of the 5 EQ-5D dimensions, of which the resulting utility values may be (slightly) different from a country tariff due to interviewer effects and modelling choices. The algorithm developed for the PRECIS can be applied in retrospect to all studies that included the PRECIS as outcome in the past.

Comparing the results of the current valuation study for the PRECIS study with the previously performed valuation study for the SCoRS [14] is challenging, because the utility scoring algorithms used different attributes and levels to construct health states. As a result, utilities derived from PRECIS and SCoRS do not represent the same health states and therefore cannot be directly compared at the health-state level. In addition, the two instruments reflect different perspectives, objective clinician evaluation versus patient’ experience, and the choice of the instrument may therefore depend on the perspective of interest.

In conclusion, cognition has been recognized as a critical dimension of schizophrenia patient health that is not adequately captured by commonly used generic preference-based measures such as the EQ-5D. In this study we demonstrated the feasibility of describing multiple domains of cognitive functioning and valuing them separately using a validated, disease-specific instrument, the PRECIS. The resulting utilities can be used to quantify improvements or deteriorations in cognitive functioning, providing a robust basis for economic evaluations. This approach aligns with the updated NICE guidance (2022), which emphasizes the importance of appropriately measuring and valuing health effects in cost-utility analyses to ensure that all relevant dimensions of health are considered. Our findings underscore the potential of cognition-specific utility measures to enhance the comprehensiveness and accuracy of health economic assessments.

## Supplementary Information

Below is the link to the electronic supplementary material.

Supplementary Material 1

## Data Availability

Data is available upon reasonable request by contacting the authors.
