# Supplementary material for: Preference-based scoring algorithm to estimate societal utilities based on the patient-reported experience of cognitive impairment in schizophrenia (PRECIS) instrument
Source: Qual Life Res. 2026 Jun 15;35(8):204. doi: 10.1007/s11136-026-04308-7 (PMC13269143; doi:10.1007/s11136-026-04308-7)
Supplement: Supplementary file 1 — Supplementary Material 1 [file 11136_2026_4308_MOESM1_ESM.pdf]

Supplementary material for manuscript

Preference-based scoring algorithm to estimate societal utilities based on the patient-reported experience of cognitive impairment in schizophrenia (PRECIS) instrument

Appendix I: Results psychometric analysis

Table A1 PRECIS Item Descriptive Statistics at Baseline Day 1 (N=215)

Item	Domain	Ceiling Effect: Lowest Score,%	Floor Effect: Highest Score,%	Missing Values,%
1.Remember Things to Do or Buy	Memory	40.93	0.47	0
2.Remember Where Things Were Put		40	1.4	0
3.Remember What to Say		41.86	1.4	0
4.Remember What Someone Else Said		34.42	3.26	0
5.Remember How to Get Somewhere		59.07	2.79	0
6.Remember What I Was About to Do		51.16	0.47	0
7.Say Something When I Wanted	Communication	44.19	3.72	0
8.Interact with People		36.74	10.7	0
9.Explaining What I Meant		43.26	4.19	0
10.Finding Words to Say What I Meant		42.79	3.26	0
11.Keep Things From Slipping Out	Self-control	51.16	2.79	0
12.Think Through Before Speaking/Doing		50.7	5.12	0
13.Stop Saying/Doing Something Wrong		59.53	2.33	0
14.Plan Ahead Without Someones Help	Executive Function	52.09	3.72	0
15.Someone Changed Plans Last Minute		40	7.91	0
16.Come Up with Solutions to Problems		46.98	3.26	0
17.Coming Up with New or Different Way		45.12	2.33	0
18.Mind Drifted Paying Attention	Attention	22.33	11.16	0
19.Distracted by My Surroundings		23.72	5.12	0
20.Hard Staying on Track		36.28	4.19	0
21.Kept Thinking About Things		34.88	8.84	0
22.Thoughts Were Racing and Speeding		43.26	6.05	0
23.Thinking was Unclear/Cloudy/Foggy		33.49	5.58	0
24.Not Thinking as Fast as Others	Sharpness of thinking	32.56	8.84	0
25.Thoughts Were Slower Than I Wanted		34.88	6.51	0
26.It Was Hard to Think What to Say		50.23	8.37	0
27.Experiences Thinking Bothersome		49.3	4.65	0
28.Bothersome if Thinking Stayed Same		35.81	11.63	0

Abbreviations: PRECIS, Patient Reported Experience of Cognitive Impairment in Schizophrenia

Table A2 Internal Consistency Reliability of PRECIS (28 item version)

	**Baseline Day 1 (n=215)**	
**PRECIS**	**Cronbach's alpha**	**Deleted item correlation with domain**
**PRECIS Total Score (28 items)**	**0.95**	
**Memory**	**0.86**	
Remember Things to Do or Buy	0.84	0.68
Remember Where Things Were Put	0.85	0.59
Remember What to Say	0.84	0.66
Remember What Someone Else Said	0.84	0.68
Remember How to Get Somewhere	0.85	0.63
Remember What I Was About to Do	0.83	0.71
**Communication**	**0.84**	
Say Something When I Wanted	0.80	0.68
Interact with People	0.84	0.62
Explaining What I Meant	0.78	0.73
Finding Words to Say What I Meant	0.79	0.72
**Self-control**	**0.79**	
Keep Things from Slipping Out	0.76	0.58
Think Through Before Speaking/Doing	0.71	0.64
Stop Saying/Doing Something Wrong	0.67	0.68
**Executive Function**	**0.85**	
Plan Ahead Without Someone's Help	0.81	0.66
Someone Changed Plans Last Minute	0.85	0.61
Come Up with Solutions to Problems	0.78	0.75
Coming Up with New or Different Way	0.78	0.74
**Attention**	**0.88**	
Mind Drifted Paying Attention	0.85	0.76
Distracted by My Surroundings	0.86	0.71
Hard Staying on Track	0.86	0.74
Kept Thinking About Things	0.87	0.66
Thoughts Were Racing and Speeding	0.88	0.61
Thinking was Unclear/Cloudy/Foggy	0.86	0.69
**Sharpness of Thinking**	**0.86**	
Not Thinking as Fast as Others	0.79	0.75
Thoughts Were Slower Than I Wanted	0.81	0.72
It Was Hard to Think What to Say	0.81	0.73

Abbreviations: PRECIS, Patient Reported Experience of Cognitive Impairment in Schizophrenia;

Table A3 Spearman's correlation Coefficients PRECIS items and EQ-5D VAS at baseline

PRECIS		EQ-5D
**Item**	**Domain**	**VAS**
		**Correlation coefficient (p-value)**
1.Remember Things to Do or Buy	Memory	-0.24 (<0.001)
2.Remember Where Things Were Put		-0.17 (0.01)
3.Remember What to Say		-0.24 (<0.001)
4.Remember What Someone Else Said		**-0.33 (<0.001)**
5.Remember How to Get Somewhere		-0.17 (0.02)
6.Remember What I Was About to Do		-0.25 (<0.001)
7.Say Something When I Wanted	Communication	-0.21 (0.002)
8.Interact with People		-0.19 (0.005)
9.Explaining What I Meant		-0.20 (0.003)
10.Finding Words to Say What I Meant		**-0.28 (<0.001)**
11.Keep Things From Slipping Out	Self-control	-0.15 (0.03)
12.Think Through Before Speaking/Doing		**-0.31 (<0.001)**
13.Stop Saying/Doing Something Wrong		-0.25 (<0.001)
14.Plan Ahead Without Someones Help	Executive Function	-0.25 (<0.001)
15.Someone Changed Plans Last Minute		-0.17 (0.02)
16.Come Up with Solutions to Problems		-0.23 (<0.001)
17.Coming Up with New or Different Way		**-0.31 (<0.001)**
18.Mind Drifted Paying Attention	Attention	**-0.26 (<0.001)**
19.Distracted by My Surroundings		-0.21 (<0.001)
20.Hard Staying on Track		-0.23 (<0.001)
21.Kept Thinking About Things		-0.15 (0.03)
22.Thoughts Were Racing and Speeding		-0.12 (0.07)
23.Thinking was Unclear/Cloudy/Foggy		-0.24 (<0.001)
24.Not Thinking as Fast as Others	Sharpness of Thinking	**-0.32 (<0.001)**
25.Thoughts Were Slower Than I Wanted		-0.28 (<0.001)
26.It Was Hard to Think What to Say		-0.24 (<0.001)

Appendix II: Final item selection

Table A4: Results psychometric analysis and expert opinion on items of the PRECIS instrument

Domain	Item	Psychometric analysis					Conclusion
		Ceiling effect (% reporting the item's best level "not at all hard")	Correlation with EQ5D VAS	EFA (loading)*	IRT (slope)*	Sum score based on normalized values#	
Memory	1.Remember Things to Do or Buy	40.9	-0.24	0.556	2.019	1.956	Item 4 selected. Clinical experts: rank 1 and 2 and highest in psychometric analyses
	2.Remember Where Things Were Put	40.1	-0.17	0.534	1.441	1.074	
	3.Remember What to Say	41.9	-0.24	0.508	2.047	1.846	
	4.Remember What Someone Else Said	34.4	-0.33	0.600	2.121	**2.754**	
	5.Remember How to Get Somewhere	59.1	-0.17	0.582	1.816	1.010	
	6.Remember What I Was About to Do	51.2	-0.25	0.831	1.691	2.062	
Communication	7.Say Something When I Wanted	44.2	-0.21	0.503	2.11	1.685	Item 10 selected. Two experts: rank 1 plus highest in
	8.Interact with People	36.7	-0.19	0.399	1.837	1.304	
	9.Explaining What I Meant	43.3	-0.2	0.604	2.546	2.290	

	10.Finding Words to Say What I Meant	42.8	-0.28	0.625	2.325	**2.531**	psychometric analyses
Self-control	11.Keep Things From Slipping Out	51.2	-0.15	0.477	1.447	0.554	Not included. Experts considered: domain less important and low scores for the psychometric analyses
	12.Think Through Before Speaking/Doing	50.7	-0.31	0.629	1.864	**2.055**	
	13.Stop Saying/Doing Something Wrong	59.5	-0.25	0.649	1.764	1.487	
Executive function	14.Plan Ahead Without Someone's Help	52.09	-0.25	0.494	2.273	1.792	Item 16 selected. Clinical experts highest rank. 2nd highest in psychometric analyses
	15.Someone Changed Plans Last Minute	40	-0.17	0.589	1.478	1.234	
	16.Come Up with Solutions to Problems	46.98	-0.23	0.772	2.237	**2.439**	
	17.Coming Up with New or Different Way	45.12	-0.31	0.770	2.125	2.765	
Attention	18.Mind Drifted Paying Attention	22.33	-0.26	0.834	2.106	**3.268**	Item 18 selected. Clinical experts high rank (2nd) and highest in psychometric analyses. Experts mentioned that item 21to 23 are not really
	19.Distracted by My Surroundings	23.72	-0.21	0.591	2.107	2.435	
	20.Hard Staying on Track	36.28	-0.23	0.743	2.26	2.681	
	21.Kept Thinking About Things	34.88	-0.15	0.591	1.531	1.328	
	22.Thoughts Were Racing and Speeding	43.26	-0.12	0.454	1.593	0.702	

	23.Thinking was Unclear/Cloudy/Foggy	33.49	-0.24	0.475	2.118	2.059	describing attention.
Sharpness of thinking	24.Not Thinking as Fast as Others	32.56	-0.32	0.672	2.31	**3.092**	Not included. Experts considered domain less important.
	25.Thoughts Were Slower Than I Wanted	34.88	-0.28	0.661	2.065	2.592	
	26.It Was Hard to Think What to Say	50.23	-0.24	0.792	1.921	2.159	

Abbreviations: EFA, Exploratory Factor Analysis; IRT, Item Response Theory; VAS, Visual Analogue Scale

*EFA and IRT results were obtained from the publication of Lenderking et al. 2024

Best performing item based on the highest sum score is presented in bold.

Appendix III DCE model results

Table A5: DCE Model estimates – multinomial logit and mixed logit model

Attribute (level)	Multinomial logit model			Mixed logit model		
	Estimate	Standard Error	P-value	Estimate	Standard Error	P-value
ASC – Choice 2	-0.0723	0.039	0.066	-0.0367	0.056	0.515
Memory (2)	-0.4769	0.097	<0.001	-0.5750	0.115	<0.001
Memory (3)	-0.7283	0.096	<0.001	-0.8673	0.106	<0.001
Memory (4)	-1.4109	0.111	<0.001	-1.7131	0.128	<0.001
Memory (5)	-2.2601	0.140	<0.001	-2.8512	0.177	<0.001
Communication (2)	-0.1714	0.099	0.085	-0.1131	0.119	0.314
Communication (3)	-0.8201	0.115	<0.001	-0.7966	0.123	<0.001
Communication (4)	-1.1028	0.116	<0.001	-1.2078	0.129	<0.001
Communication (5)	-2.3842	0.165	<0.001	-2.9655	0.187	<0.001
Executive Functioning (2)	-0.7014	0.095	<0.001	-0.8272	0.112	0.006
Executive Functioning (3)	-1.0161	0.098	<0.001	-1.2514	0.117	0.005
Executive Functioning (4)	-1.4609	0.087	<0.001	-1.8507	0.125	<0.001
Executive Functioning (5)	-2.2497	0.151	<0.001	-2.8907	0.186	<0.001
Attention (2)	-0.1849	0.094	0.050	-0.3243	0.119	0.006
Attention (3)	-0.1695	0.093	0.069	-0.3428	0.123	0.005
Attention (4)	-0.6088	0.104	<0.001	-0.9017	0.129	<0.001
Attention (5)	-1.4297	0.125	<0.001	-1.9910	0.187	<0.001
sd.memory (2)				0.8021	0.205	<0.001
sd.memory (3)				-0.3446	0.212	0.105
sd.memory (4)				-0.055	0.257	0.831
sd.memory (5)				0.8125	0.169	<0.001
sd.communication (2)				0.3504	0.185	0.058
sd.communication (3)				0.162	0.275	0.556
sd.communication (4)				-0.3999	0.193	0.038
sd.communication (5)				1.1141	0.184	<0.001
sd.execfunct (2)				0.1495	0.334	0.654
sd.execfunct (3)				0.0597	0.261	0.819
sd.execfunct (4)				-0.0767	0.220	0.728
sd.execfunct (5)				1.2414	0.164	<0.001
sd.attention (2)				0.3215	0.215	0.135
sd.attention (3)				-0.0598	0.296	0.840
sd.attention (4)				-0.1517	0.238	0.524
sd.attention (5)				0.8996	0.228	<0.001
AIC			3940.82			3894.38
BIC			4051.61			4132.32

Adjusted Pseudo R^2	0.4316	0.4380
Likelihood	-1953.408	-1914.19
DF	17	33
LR Chisq (P-value)	78.436 (<0.001)	

Note: Level 1 of each attribute is the reference level "not at all/not at all hard"
(2) a little bit/a little bit hard, (3) somewhat/somewhat hard, (4) quite a bit/quite hard, (5) very much/very hard

Abbreviations: ASC, Alternative-Specific Constant; AIC, Akaike Information Criterion; BIC, Bayesian Information Criterion; DF, Degree of Freedom; LR, Likelihood Ratio; sd, standard deviation (estimated standard deviation of the random parameter distribution for each level)
